# Bi-directional relationships between physical activity and mental health among a large sample of Canadian youth: a sex-stratified analysis of students in the COMPASS study

**DOI:** 10.1186/s12966-021-01201-z

**Published:** 2021-10-09

**Authors:** M. Claire Buchan, Isabella Romano, Alexandra Butler, Rachel E. Laxer, Karen A. Patte, Scott T. Leatherdale

**Affiliations:** 1 School of Public Health Sciences, University of Waterloo, 200 University Avenue West, Waterloo, Ontario N2L 3G1 Canada; 2 Public Health Ontario, Toronto, Ontario Canada; 3 Department of Health Sciences, Brock University, St. Catharines, Ontario Canada

**Keywords:** Physical activity, Depression, Anxiety, Adolescent, Cross-lagged model

## Abstract

**Objective:**

The aim of this research was to examine the bidirectional association between self-reported symptoms of mental disorder and physical activity among a large sample of Canadian secondary school students over time.

**Methods:**

Linked survey data were obtained from 28,567 grade 9 to 12 students across Canada participating in two waves of the COMPASS Study (2017–18; 2018–19). Autoregressive cross-lagged models were run to examine the reciprocal relationships between self-reported moderate-to-vigorous physical activity (MVPA) and symptoms of depression (CESD-10) and anxiety (GAD-7). Models were stratified by gender, and accounted for grade, ethno-racial identity, and school-level clustering.

**Results:**

Autoregressive associations show that neither symptoms of anxiety nor depression, at baseline, were predictive of mean MVPA at follow-up – consistent for the full sample and among both males and females. Higher MVPA among males at baseline was associated with lower symptoms of both anxiety (β = − 0.03, *p* = 0.002) and depression (β = − 0.05, *p* < 0.001) at follow-up. However, among females, higher MVPA at baseline was associated with greater symptoms of anxiety (β = 0.03, *p* < 0.001), but not symptoms of depression (β = 0.01, *p* = 0.073), at follow-up.

**Conclusion:**

In our large sample of Canadian secondary school students, associations between physical activity and symptoms of mental disorder were not bi-directional, and these relationships differed in males and females. This study illustrates the complex nature of the relationship between physical activity and symptoms of mental disorder among youth. While results support the benefits of promoting physical activity among males to prevent or manage internalizing symptoms, the relationship among females warrants further investigation.

**Supplementary Information:**

The online version contains supplementary material available at 10.1186/s12966-021-01201-z.

## Introduction

Mental disorders and physical inactivity are two leading causes of poor health outcomes and disability later in life in Canada [1–3]. Symptoms of depression and anxiety often begin in childhood and track into adulthood, with nearly 20% of Canadian youth meeting the criteria for at least one mental disorder diagnosis prior to the age of 18 [4–6]. Without appropriate support and treatment, poor mental health can lead to a variety of adverse health outcomes including poor academic outcomes, substance use, self-harm, and suicidal behaviour [7–9]. Similarly, time spent engaging in moderate-to-vigorous physical activity (MVPA) tends to decrease throughout adolescence, and less than 50% meet recommended guidelines for daily MVPA [10]. There is an established link between MVPA and mental health in both adult and youth populations [11–16], and physical activity is recommended as a first-line treatment for mild-to-moderate depression in clinical samples of adults [17, 18]. While some literature indicates physical activity may be an opportunity for early and cost-effective intervention and a population-based approach [19] to promote mental health, evidence on this relationship remains less documented in youth [20].

Most of the research examining the association between MVPA and symptoms of depression and anxiety among youth to date has been cross-sectional with only a few examining the longitudinal effects [21–23]. For example, physical activity has been found to be positively associated with mental health outcomes, including lower symptoms of depression [22, 24] and higher physical self-concept [22], and there is some evidence for a small-to-moderate positive effect on anxiety [25]. On the other hand, those experiencing poorer mental health may be less engaged in physical activity. Youth diagnosed with psychiatric disorders are more likely to report low levels of physical activity [26, 27], while those reporting higher confidence and stronger social networks, as well as fewer symptoms of depression, are more likely to participate in organized sport [21]. Involvement in sport also seems to enhance confidence, strengthen networks, and reduce symptoms of depression among youth participants [21]. Studies summarized by Eime et al. [21] did not examine the directionalily of the relationship between physical activity, depression, and anxiety, however their findings suggest that the relationship between psychosocial wellbeing and sport participation may in fact be bidirectional. Longitudinal research examining physical activity and mental health among youth is limited and inconsistent [23, 28]. Although some research suggests that changes in physical activity over time may predict depression [20, 29, 30], others have found no significant association [31, 32].

The relationship between physical activity and mental health may have differential effects for males and females [26, 33]. Previous research demonstrates that frequency of physical activity and fitness capabilities were both negatively correlated with internalizing disorders and psychosocial problems, however, this relationship was stronger for males than females [33]. The protective effects of physical activity on mental health may be sustained over time, although this was observed within males only [34]. Other research has identified a modest favourable association between fitness and internalizing problems only among females [35, 36]. Moreover, higher internalizing scores have also been shown to reduce physical activity among males in particular, but not females [26]. Additional evidence is required to delineate the sex- and gender-based effects of physical activity on mental health, and vice versa, in adolescents.

While there is some evidence indicating that physical activity and mental health may be bidirectionally related, most of the research examining directionality has been conducted within adult populations [11–14]. Findings from these studies generally support the hypothesis that engaging in physical activity tends to improve mental health, and likewise, those with better self-reported mental health tend to engage in adequate amounts of physical activity. One study that examined bidirectional relationships among a small sample of Canadian youth identified an independent relationship between mental health and physical activity, while bidirectional relationships were largely null [28]. However, this study used a relatively small sample of adolescents from one region in Canada and did not examine sex-based differences in this relationship.

Finding ways to improve both physical activity and symptoms of depression and anxiety among youth should be a priority for public health. The high prevalence of, and potentially severe outcomes associated with poor psychological functioning in adolescence, demonstrate the importance of identifying cost-effective and population-based approaches to promote mental health, including physical activity. To make such recommendations, it is important to better understand the complexity and interrelationships between physical activity and mental health among youth and how these relationships may differ by sex. Among a large sample of Canadian secondary school students, the primary objective of this study was to examine the sex-stratified, bidirectional association between self-reported anxiety and depressive symptoms with self-reported physical activity. Our secondary objective was to compare sex-based associations by examining female and male students separately. Based on the aforementioned evidence and the study population under evaluation, it is expected that bidirectional relationships will be observed. Specifically, we hypothesize that physical activity and symptoms of anxiety and depression will be stable over time and that higher physical activity will be associated with lower symptoms of anxiety and depression and vice versa, 1 year later.

## Methods

### Study design

The COMPASS Study (2012–2021) is a prospective cohort study collecting health behaviour data from students attending a convenience sample of secondary schools across Canada (i.e., grades 9–12 in Alberta, British Columbia, Ontario; secondaire I-V in Quebec) [37]. Schools were purposefully recruited based on the permitted use of active-information passive-consent permission protocols, which are critical for collecting robust youth data on mental health [38]. All students attending participating schools were eligible to participate and can withdraw at any time. The COMPASS student questionnaire (Cq) is a paper-and-pencil survey completed by full school samples once annually during class time. A full description of the COMPASS Study and its methods can be found elsewhere in print [37] and online (www.COMPASS.uwaterloo.ca). All procedures were approved by the University of Waterloo Office of Research Ethics (ORE: 30118) and participating school boards.

### Participants

The current study used linked data from 28,567 secondary school students participating in two waves of the COMPASS Study (Time 1 (T1): 2017–18; Time 2 (T2): 2018–19). The COMPASS mental health module was piloted in 2016/17, therefore the waves of data included in this analysis reflect the years in which mental health data were collected for the entire cohort. Students represented a convenience sample of 117 secondary schools (14 in British Columbia, 8 in Alberta, 59 in Ontario, 36 in Quebec) that participated over both waves of data collection. Student data were linked using a unique, self-generated code as described in COMPASS data collection protocols [39]. Nonlinkage primarily resulted from student absence on one of the two data collection dates (e.g., due to school field trips, sports events, etc.).

### Measures

Based on the proposed objectives, the specific student-level items to be examined in this research included self-reported moderate-to-vigorous physical acitivity (MVPA) and symptoms of depression and anxiety. On the Cq, students were asked to mark how many minutes of: (a) moderate physical acitivity, and (b) hard physical activity they engaged in on each of the last 7 days [in hours (0 to 4) and minutes (0, 15, 30, 45)]. Daily MVPA was computed using the combined total minutes of ‘moderate’ and ‘hard’ intensity physical activity weekly, divided by seven. Based on the Canadian 24-Hour Movement Guidelines [40], students were classified as meeting the MVPA guidelines if they had performed an average of 60 min of MVPA daily. Self-reported MVPA in the Cq has demonstrated satisfactory reliability and validity among youth [41].

Youth depressive symptoms were assessed using *the Center for Epidemiologic Studies Depression (Revised) 10-item scale* (CESD-R-10 [42];). The CESD-R-10 includes 10 items on positive and negative affect, anhedonia, somatic symptoms, and perceptions on relationships; students were asked to rate the frequency of symptoms they experienced in the last week. Self-reported anxiety symptoms were assessed using the *Generalized Anxiety Disorder 7-item scale* (GAD-7 [43];). The GAD-7 measures difficulty controlling feelings of worry, nervousness, restlessness, and irritability over a 2-week period of time. Both scales have been validated for use in adolescent populations [43–45]. A four-point Likert scale is used to score each item in both the CESD-R-10 and GAD-7, with higher scores indicating greater risk of generalized anxiety and major depression. Total scores for the CESD-R-10 range from 0 to 30 and total scores for the GAD-7 range from 0 to 21. For both scales, clinically-relevant cut-off values for major depression [45, 46] and generalized anxiety [47, 48] are represented by scores equal to or greater than 10 (≥10). The internal consistency reliability (Cronbach’s α) for the CESD-R-10 and GAD-7 measures were high in our sample (α_CESD-R-10_ = 0.96; α_GAD-7_ = 0.95).

The Cq also collects demographic information including grade (9, 10, 11, 12, other^1^), sex (males, females), and students’ ethno-racial identity (defined as non-racialized [White] or racialized [Black, Asian, Latin American or Hispanic, Mixed, Other]). Students also reported their weekly spending/saving money from allowance or part-time employment ($0, $1–20, $20–100, $100+, don’t know), which was considered a proxy for student-level socioeconomic status as this is a more accessible value for youth to report on than household income [49].

### Analysis

Autoregressive cross-lagged (ARCL) models were fit to examine bidirectional associations between mean daily MVPA and CESD-R-10 and GAD-7 sum scores at T1 and T2 among the full sample of students and stratified by sex. Compared to traditional analytic approaches, the ARCL models allowed for the simultaneous assessment of whether health outcomes were predictive of themselves as well as other health outcomes over time by combining two modeling strategies [50]. The ‘autoregressive’ model first estimated whether subsequent health outcomes at T2 were predicted by the same health outcome at T1 (i.e., mean daily MVPA at T1 predicts mean daily MVPA at T2 follow up). Less robust cross-lagged correlation analyses do not account for the stability of constructs over time (Kearney, 2017), and the autoregressive component of the ARCL model addresses this limitation. Second, the ARCL model estimated whether a subsequent health outcome (e.g., CESD-R-10 sum score at T2) was predicted by a different health outcome at an earlier time (e.g., mean daily MVPA at T1) and vice versa. All models accounted for grade, ethno-racial identity, and school-level clustering. Mean daily MVPA and CESD-R-10 and GAD-7 sum scores were modelled continuously and beta estimates were reported alongside 95% confidence limits. Model fit was assessed using root mean square error of approximation (RMSEA), the comparative fit index (CFI) and Tucker-Lewis index (TLI). A RMSEA value closer to zero, and CFI and TLI values that are closer to 1, indicate better model fit [51]. We also reported the Chi-square goodness-of-fit index, but did not rely on it as it is highly influenced on sample size.

We used Mplus 8.0 [52] software to fit the ARCL model with full information maximum likelihood (FIML) to retain cases with missing outcome data (mean daily MVPA, and CESD-R-10 amd GAD-7 sum scores) [52]. At T1, rates of missingness for each outcome were as follows: MVPA (*n* = 448, 1.6%), CESD-R-10 score (*n* = 3838, 13.4%]), and GAD-7 score (*n* = 1910, 6.7%). At T2, 1.6% of students had missing MVPA data (*n* = 455), 11.2% had missing CESD-R-10 scores (*n* = 3212), and 6.0% had missing GAD-7 scores (*n* = 1726). Additional analyses predicting missingness across outcome variables at T1 and T2 are presented in Supplementary Table A.

## Results

**Fig. 1:**
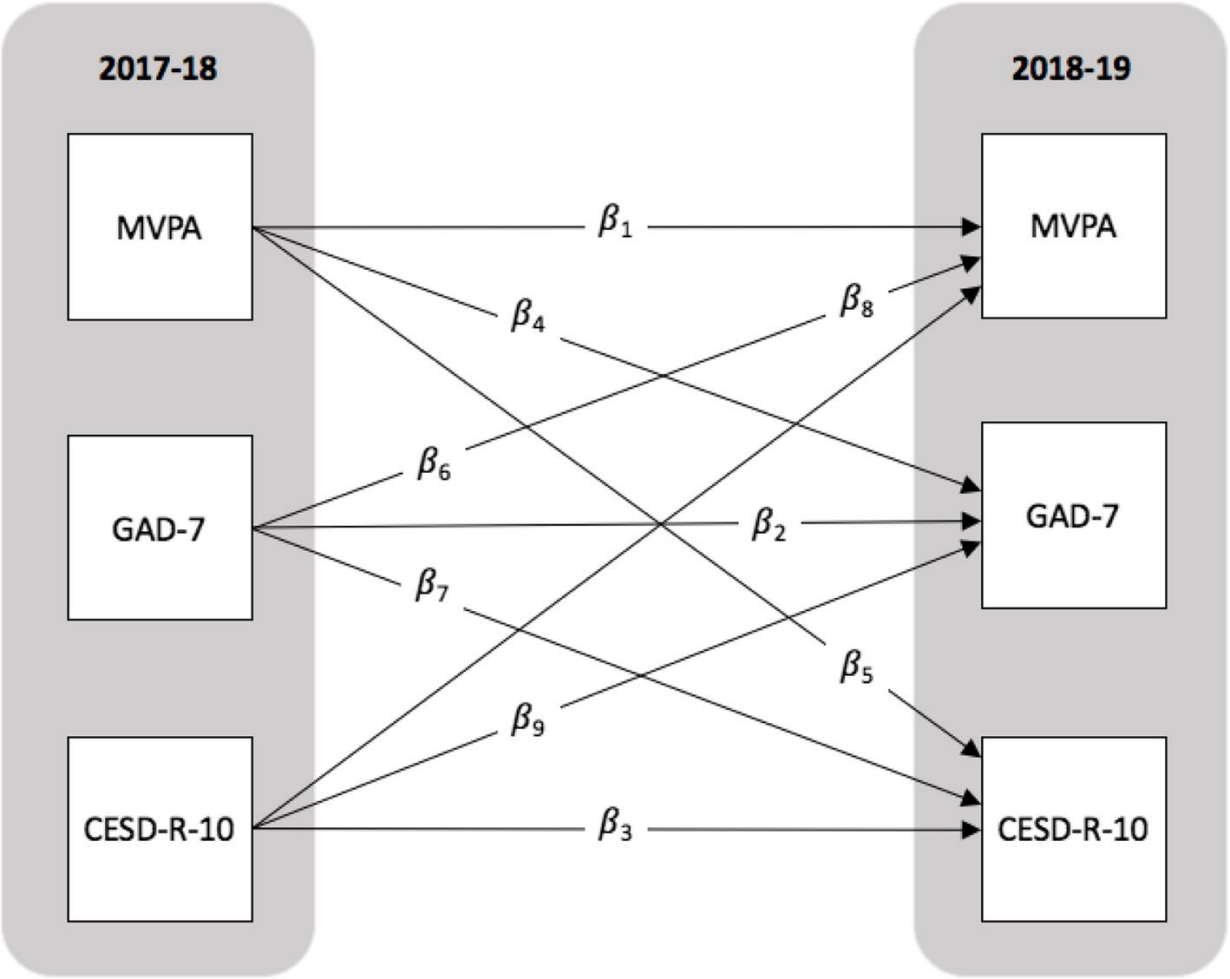
Structural diagram depicting autoregressive and cross-lagged associations between mean daily moderate-to-vigorous physical activity (MVPA), and anxiety (GAD-7) and depression (CESD-R-10) symptom scores, among adolescents who participated in the 2017–18 and 2018–19 school years of the COMPASS Study in Alberta, British Columbia, Ontario, and Quebec. *Note*. β1- β9 correspond with estimates listed in Table 3

Baseline (2017–18) characteristics among students in our analytic sample are presented by sex in Table 1. Students’ mean rates of daily MVPA, and CESD-R-10 and GAD-7 sum scores are shown by COMPASS study year and sex in Table 2; the proportion of students who reported meeting daily MVPA guidelines and clinically-relevant CESD-R-10 and GAD-7 scores are also reported in Table 2. Corresponding to Fig. 1, Table 3 presents the ARCL associations between mean daily MVPA and CESD-R-10 and GAD-7 sum scores among students (Model I: full sample of students, Model II: females only, Model III: males only) who participated in the 2017–18 and 2018–19 school years of the COMPASS study.

**Table 1:** Descriptive comparisons of students’ baseline (2017–18) characteristics by sex, among adolescents who participated in the 2017–18 and 2018–19 school years of the COMPASS Study in Alberta, British Columbia, Ontario, and Quebec

Measure	Females	Males	
	*n* (%)	*n* (%)	χ^2^, *t* (*p*)
**Grade [age, in years]**			
9 (*ref.*) [14–15]	3972 (31.8)	3522 (32.3)	45.8 (< 0.0001)
10 [15–16]	3923 (31.4)	3352 (30.8)	
11 [16–17]	2373 (19.0)	2109 (19.4)	
12 [17–18]	82 (0.7)	163 (1.5)	
Other	2152 (17.2)	1747 (16.0)	
**Race/ethnicity**			
Non-racialized (*ref.*)	9860 (78.9)	8464 (77.7)	4.7 (0.0308)
Racialized	2642 (21.1)	2429 (22.3)	
**Weekly spending money**			
$0 or ‘don’t know’ (*ref*.)	4375 (35.0)	3927 (36.3)	25.3 (< 0.0001)
$1–20	3676 (29.4)	3092 (28.6)	
$21–100	2837 (22.7)	2278 (21.0)	
$100+	1536 (12.3)	1524 (14.1)	
**Province**			
Alberta (*ref*.)	643 (5.1)	607 (5.6)	22.4 (< 0.0001)
British Columbia	1657 (13.3)	1527 (14.0)	
Ontario	5921 (47.4)	5341 (49.0)	
Québec	4281 (34.2)	3418 (31.4)	

*Note. ref.* reference category

**Table 2:** Mean rates of daily moderate-to-vigorous physical activity, and anxiety and depression symptom scores, among adolescents who participated in the 2017–18 and 2018–19 school years of the COMPASS Study in Alberta, British Columbia, Ontario, and Quebec

Measure	2017–18, *n* (%)				2018–19, *n* (%)			
	Full sample	Females only	Males only	χ^2^, *t* (*p*)	Full sample	Females only	Males only	χ^2^, *t* (*p*)
**Physical activity**								
Daily MVPA minutes, mean (SD)	107.9 (78.6)	98.5 (72.0)	118.7 (84.2)	−19.6 (< 0.0001)	101.9 (77.4)	91.8 (70.1)	113.7 (83.6)	−12.8 (< 0.0001)
Meets MVPA guidelines	9300 (39.8)	4129 (33.0)	5171 (47.5)	507.1 (< 0.0001)	8888 (37.0)	3926 (30.3)	4962 (44.9)	540.0 (< 0.0001)
Does not meet MVPA guidelines	14,095 (60.2)	8373 (67.0)	5722 (52.5)		15,119 (63.0)	9019 (69.7)	6100 (55.1)	
**Anxiety symptoms**								
GAD-7 score, mean (SD)	5.9 (5.4)	7.3 (5.7)	4.3 (4.6)	44.4 (< 0.0001)	6.5 (5.6)	8.0 (5.7)	4.8 (4.9)	47.5 (< 0.0001)
GAD-7 score ≥ 10	5150 (22.0)	3786 (30.3)	1364 (12.5)	1069.7 (< 0.0001)	6189 (25.8)	4482 (34.6)	1707 (15.4)	1148.3 (< 0.0001)
GAD-7 score < 10	18,245 (78.0)	8716 (69.7)	9529 (87.5)		17,818 (74.2)	8463 (65.4)	9355 (84.6)	
**Depression symptoms**								
CESD-R-10 score, mean (SD)	8.1 (5.8)	9.3 (6.2)	6.8 (4.9)	33.4 (< 0.0001)	9.0 (6.0)	10.2 (6.3)	7.5 (5.4)	35.8 (< 0.0001)
CESD-R-10 score ≥ 10	7470 (31.9)	5024 (40.2)	2446 (22.5)	842.0 (< 0.0001)	9241 (38.5)	6180 (47.7)	3061 (27.7)	1014.7 (< 0.0001)
CESD-R-10 score < 10	15,925 (68.1)	7478 (59.8)	8447 (77.5)		14,766 (61.5)	6765 (52.3)	8001 (72.3)	

*Note*. *MVPA* moderate-to-vigorous physical activity, *SD* standard deviation

**Table 3:** Standardized model parameter estimates, stratified by sex, predicting the autoregressive and cross-lagged associations between mean daily moderate-to-vigorous physical activity, and anxiety and depression symptom scores, among adolescents who participated in the 2017–18 and 2018–19 school years of the COMPASS Study in Alberta, British Columbia, Ontario, and Quebec

Parameter Estimate^a^	Model I			Model II			Model III		
	Full sample			Females only			Males only		
	β	95% CL	*p*	β	95% CL	*p*	β	95% CL	*p*
**Autoregressive**									
β_1_ (MVPA)	0.53	0.51, 0.54	< 0.001	0.53	0.51, 0.55	< 0.001	0.52	0.50, 0.55	< 0.001
β_2_ (GAD-7)	0.47	0.45, 0.49	< 0.001	0.48	0.45, 0.50	< 0.001	0.43	0.39, 0.46	< 0.001
β_3_ (CESD-R-10)	0.47	0.45, 0.49	< 0.001	0.50	0.47, 0.53	< 0.001	0.41	0.38, 0.44	< 0.001
**Cross-lagged**									
β_4_ (MVPA → GAD-7)	0.00	− 0.01, 0.01	0.739	0.03	0.02, 0.05	< 0.001	− 0.03	−0.05, − 0.01	0.002
β_5_ (MVPA → CESD-R-10)	−0.02	− 0.03, − 0.01	0.002	0.01	0.00, 0.03	0.073	−0.05	− 0.07, − 0.04	< 0.001
β_6_ (GAD-7 → MVPA)	−0.01	− 0.03, 0.01	0.499	0.00	−0.03, 0.03	0.944	−0.02	− 0.05, 0.02	0.317
β_7_ (GAD-7 → CESD-R-10)	0.18	0.16, 0.20	< 0.001	0.15	0.12, 0.18	< 0.001	0.21	0.17, 0.24	< 0.001
β_8_ (CESD-R-10 → MVPA)	0.01	−0.02, 0.03	0.640	0.01	−0.02, 0.04	0.507	0.00	−0.03, 0.03	0.881
β_9_ (CESD-R-10 → GAD-7)	0.18	0.16, 0.20	< 0.001	0.18	0.15, 0.20	< 0.001	0.18	0.15, 0.21	< 0.001

^a^Adjusted for grade, race/ethnicity, and school clustering

Autoregressive associations show measures of MVPA, CESD-R-10, and GAD-7 were stable over time – consistent for the full sample and among both males and females. Among the full sample (Model I), a positive association was identified between mean GAD-7 score in 2017–18 and mean CESD-R-10 score in 2018–19 (β = 0.18, 95% CL [0.16, 0.20]). This association was reciprocal, whereby mean CESD-R-10 score in 2017–18 also predicted mean GAD-7 score in 2018–19 (β = 0.18, 95% CL [0.16, 0.20]). However, GAD-7 and CESD-R-10 scores in 2017–18 were not predictive of mean MVPA in 2018–19. Rather, higher mean daily MVPA were associated with lower CESD-R-10 scale scores at follow-up in 2018–19 (β = − 0.02, 95% CL [− 0.03, − 0.01]), but not significantly associated with GAD-7 scores.

Among females (Model II), the cross-lagged associations between CESD-R-10 and GAD-7 scores were also reciprocal; higher CESD-R-10 sum scores in 2017–18 were associated with higher GAD-7 scores in 2018–19 (β = 0.18, 95% CL [0.15, 0.20]) and vice versa (β = 0.15, 95% CL [0.12, 0.18]). Similar to the full sample, neither GAD-7 nor CESD-R-10 scores in 2017–18 were predictive of mean MVPA in 2018–19 for females. Higher MVPA among females in 2017–18 was associated with higher mean GAD-7 score (β = 0.03, 95% CL [0.02, 0.05]) in 2018–19, but was not associated with mean CESD-R-10 score. Results among males (Model III) also indicated a significant and cross-lagged association between GAD-7 score in 2017–18 and CESD-R-10 in 2018–19 (β = 0.21, 95% CL [0.17, 0.24]) and vice versa (β = 0.18, 95% CL [0.15, 0.21]). Baseline GAD-7 and CESD-R-10 scores were not associated with mean MVPA at follow-up. However, among males, higher MVPA in 2017–18 was associated lower mean GAD-7 (β = − 0.03, 95% CL [− 0.05, − 0.01]) and CESD-R-10 (β = − 0.05, 95% CL [− 0.07, − 0.04]) scores in 2018–19.

Model fit statistics are presented in Table 4 by strata. Results demonstrate excellent overall fit across all indices (i.e., CFI, TLI, RMSEA) for the sex-specific ARCL models; model fit is also presented for the full sample. Effect sizes in this study appear small, however they represent scores along continuous variables with small unit increases (e.g., for every 1-min increase in average MVPA, there was a 3% increase in GAD-7 score among females – meaning a 10 min difference translates to a 30% increase in anxiety symptoms). When taken into context of the variables being modelled, these findings represent quite dramatic effects, especially at the population level.

**Table 4:** Fit statistics for models predicting the autoregressive and cross-lagged associations between mean daily moderate-to-vigorous physical activity, and anxiety and depression symptom scores, among adolescents who participated in the 2017–18 and 2018–19 school years of the COMPASS Study in Alberta, British Columbia, Ontario, and Quebec

Index^a^	Model I	Model II	Model III
	Full sample	Females only	Males only
CFI	0.959	0.992	0.991
TLI	0.904	0.997	0.972
RMSEA (90% CI)	0.090 (0.085, 0.094)	0.046 (0.039, 0.054)	0.046 (0.039, 0.054)
Chi-square (*p*)	29,238.754 (< 0.0001)	15,159.439 (< 0.0001)	10,792.477 (< 0.0001)

For the RMSEA, values closer to 0 indicate better fit

^a^For the CFI and TLI, values closer to 1 indicate better fit

## Discussion

This study sought to investigate bidirectional associations between physical activity and symptoms of depression and anxiety in a large sample of secondary school youth in Canada. To the best of our knowledge, this is the first study to examine sex differences in the reciprocal relationship between physical activity and symptoms of depression and anxiety among youth. Our findings indicated that MVPA, and symptoms of depression and anxiety all demonstrated stability over time in our sample; that is, there was minimal variance in individual levels of MVPA, symptoms of depression and anxiety over 1 year. Cross-lagged analyses suggested that the relationships between MVPA and symptoms of depression and anxiety differed in females and males in our sample. Greater MVPA at baseline was associated with lower symptoms of both anxiety and depression among males, but with greater symptoms of anxiety among females, at 1 year follow-up. Conversely, neither symptoms of anxiety or depression were associated with levels of MVPA among males or females at follow-up. Symptoms of depression and anxiety, however, were predictive of each other. These results highlight the importance of exploring sex differences in research on physical activity and mental health, and that the relationship that exists is not bidirectional. Previous research in this area has demonstrated that physical activity – either measured through volume of MVPA [24, 32, 53–56] or sport participation [32, 57] – has the potential to reduce the risk of depression among youth; few studies have reported results stratified by sex [33, 34]. Aside from one study among youth that identified a bidirectional relationship between symptoms of depression and physical activity over time [28], all other analyses have investigated the bidirectional nature of this relationship among varying samples of adults [12, 13, 58, 59]; only two of these identified studies examined how these relationships differ by sex [11, 14]. Interestingly, neither reported significant differences between men and women. Given that our findings vary from those seen among samples of adults, it is possible that sex-based differences are more pronounced during adolescence, where puberty and developmental timing may play a role; further research is needed to confirm these age-related differences.

These results are consistent with those from a previous longitudinal study that found volume of physical activity to be negatively associated with emotional and peer problems in males, but not females [34]. Preliminary evidence suggests that there may be sex differences in the intention behind physical activity, with females engaging in physical activity primarily for weight and body image reasons, and males engaging primarily for social recognition, enjoyment, and strength training [60]. It is therefore possible that the negative impacts of physical activity on symptoms of anxiety among females within our sample could be attributed to concerns surrounding body image and pressure to achieve weight or physical appearance goals. We speculate that males may participate in physical activity for enjoyment and overall health purposes and as such, experience many of the positive benefits of becoming more active including lower symptoms of anxiety and depression.

Within the context of other available literature, we suspect that type of activity would partially explain the sex-based differences observed in the relationship between physical activity and mental health outcomes. Previous research suggests that youth who engaged in physical activity through a sports club had lower odds for symptoms of depression than those who participated in other physical activity contexts [57]. Team sports have been associated with lower levels of anxiety [61], depression [61, 62] and overall mental health [63] than individual sports. Further, individual sport athletes have been found more likely to participate in sport for goal-oriented reasons, rather than for enjoyment purposes [61]. Research has shown that a larger proportion of males often opt to participate in team sports, and a larger proportion of females opt to participate in individual sports [64]. Team sports provide an opportunity for socialization and emotional development [21], which could partially explain the way in which physical activity positively influences symptoms of mental health. This suggests that the social interaction associated with sports clubs and organized activities in addition to the physical activity itself could result in a reduction of depressive symptoms in youth. This relationship and associated outcomes warrant further investigation.

In contrast to previous research, symptoms of depression or anxiety were not found to predict physical activity levels in this large sample of school-aged youth [28]. However, our results indicate consistent bidirectional relationships between depressive and anxiety symptoms over time within the full sample and among both males and females. These findings align with previous literature that highlights the comorbid nature of internalizing mental disorders. Youth reporting symptoms of anxiety are more likely to report subsequent symptoms of depression, and vice versa [65, 66]. Studies exploring the impacts of depression and anxiety on MVPA to date have been mixed [14, 67, 68]. Overall, our findings suggest that symptoms of depression or anxiety are not predictive of MVPA over 1 year; however, it is possible that a relationship may be present if students were tracked longitudinally through elementary and secondary school, or if the impact of changes in mental disorder over time on physical activity levels was examined. Further, these relationships may be more pronounced in populations that report more severe symptoms of mental disorder or a clinical diagnosis. Despite the statistical non-significance observed in our study, previous research supports the notion that physical inactivity should still be considered a potential long-term consequence of depression and anxiety and warrants further investigation [29, 68].

In light of our results, understanding if physical activity setting, intensity, or type impact this relationship might provide further context and direction for prevention efforts among youth. Moreover, a deeper exploration of the sex-based differences identified in this study as well as potential effect modifiers, such as weight perception, resiliency, or motivation behind physical activity is necessary. Future research should also consider the negative impacts of sporting or physical activity as they relate to sex-based contexts; often, these are oriented toward males and not females, even within school environments. Physical activity has the potential to offer youth an opportunity to develop new skills, foster social supports, and enhance their self-esteem and self-concept, all of which could contribute to reductions in depressive symptoms [69]. However, a better understanding of the effectiveness of physical activity promotion among females to mitigate disproportionate impacts is imperative. Once better understood, physical activity programming could have important implications for primary prevention in youth mental disorder. Physical activity interventions have the ability for large impact, and are typically low risk and cost effective, especially when delivered through the school context [70].

### Strengths and limitations

Our findings must be considered in the context of the following limitations. Firstly, despite being a large sample of secondary school-aged youth, our sample is not representative of all adolescents in Canada. COMPASS uses a convenience sampling approach and therefore our results may not be generalizable to all school-aged youth in Canada. Second, we used self-reported symptoms rather than clinical diagnoses to detect changes in depression and anxiety. Third, we did not control for sedentary behaviour in our analyses due to strong ceiling effects present within our sample. Future studies should account for sedentary behaviour when feasible. Fourth, due to the limited follow-up data (i.e., only 1 year of follow-up), it is not possible to determine causality. Future studies should consider using multiple or longer follow-up times to determine if potentially more complex relationships take longer to become apparent. Examining trajectories of physical activity levels and mental health outcomes could provide greater insight on the temporality and/or causality of this relationship. Finally, data for this study were collected using self-report questionnaires. Self-report data may result in an overestimation of true physical activity levels often due to recall or social desirability bias [71], as such, our results may underestimate the true effect of physical activity on mental health outcomes. The questionnaires used in the COMPASS study, however, have been previously validated as reliable measures of physical activity [41] and mental health [72] in youth. While our proxy measure of SES has been previously used in research among youth, it is possible that youth from high SES households may report lower weekly spending money as they may not be required to have a part-time job. Given our sizeable sample, it is possible that the analyses in this study were overpowered. While we report both effect sizes and *P*-values, we caution readers from interpreting our findings using *P*-values alone.

## Conclusion

This study examined the sex-based and bidirectional relationship between physical activity and symptoms of depression and anxiety among youth. Our findings indicated that in our large sample of Canadian secondary school students, associations between physical activity and symptoms of depression and anxiety were not bi-directional. When stratified by sex, higher MVPA was associated with decreases in both anxiety and depression symptoms among males; and higher MVPA was associated with elevated symptoms of anxiety among females. This study illustrates the complex nature of the relationship between physical activity and symptoms of mental disorder among youth. While results support the benefits of promoting physical activity among males to prevent or manage symptoms of depression and anxiety, the relationship among females warrants further investigation. Future research should explore how intentions behind physical activity, weight perception and control, or type and duration of physical activity influence these associations. These findings should be taken into consideration when developing youth physical activity programming, particularly when delivered through the school context.

## Supplementary Information

**Additional file 1: Supplementary Table A.** Logistic regression model predicting likelihood of missing outcome data among adolescents who participated in the 2017–18 and 2018–19 school years of the COMPASS Study in Alberta, British Columbia, Ontario, and Quebec.

## Data Availability

The datasets generated and analyzed during the current study will not currently be shared because this is an ongoing study; however, access to the data supporting the findings of the study can be requested at https:// uwaterloo.ca/compass-system/information-researchers.

## Footnotes

1 Students who did not fall within grades 9–12 were categorized as ‘other.’ These included students in Québec enrolled in Secondaire I and II (equivalent to grades 7 and 8 in other provinces) as well as those enrolled in a secondary school class with no official grade equivalent (e.g., “new immigrant” classes in Québec). Note there is no grade 12 in Québec.
