# Supplementary material for: Bi-directional relationships between physical activity and mental health among a large sample of Canadian youth: a sex-stratified analysis of students in the COMPASS study
Source: Int J Behav Nutr Phys Act. 2021 Oct 9;18:132. doi: 10.1186/s12966-021-01201-z (PMC8501578; doi:10.1186/s12966-021-01201-z)
Supplement: Supplementary file 1 — Additional file 1: Supplementary Table A. Logistic regression model predicting likelihood of missing outcome data among adolescents who participated in the 2017–18 and 2018–19 school years of the COMPASS Study in Alberta, British Columbia, Ontario, and Quebec. [file 12966_2021_1201_MOESM1_ESM.docx]

| **Supplementary Table A.** Logistic regression model predicting likelihood of missing outcome data among adolescents who participated in the 2017-18 and 2018-19 school years of the COMPASS Study in Alberta, British Columbia, Ontario, and Quebec. | | | | | | |
| --- | --- | --- | --- | --- | --- | --- |
|  | **2017-18**, aOR (95% CI) | | | **2018-19**, aOR (95% CI) | | |
| Measure | I | II | III | I | II | III |
| **Sex** |  |  |  |  |  |  |
| Male (*ref.*) | 1.00 | 1.00 | 1.00 | 1.00 | 1.00 | 1.00 |
| Female | 1.00 (0.82-1.22) | 0.94 (0.87-1.01) | 1.04 (0.94-1.15) | 0.83 (0.69-0.99)* | 0.83 (0.77-0.90)** | 0.86 (0.78-0.94)** |
| **Grade** |  |  |  |  |  |  |
| 9 (*ref.*) | 1.00 | 1.00 | 1.00 | 1.00 | 1.00 | 1.00 |
| 10 | 1.30 (0.96-1.75) | 1.48 (1.29-1.71)** | 1.46 (1.23-1.72)** | 1.37 (0.92-2.03) | 1.27 (1.08-1.49)** | 1.04 (0.80-1.34) |
| 11 | 1.28 (0.59-2.77) | 0.82 (0.60-1.13) | 0.98 (0.58-1.64) | 0.69 (0.41-1.14) | 0.76 (0.63-0.92)** | 0.80 (0.61-1.05) |
| 12 | 0.79 (0.59-1.05) | 0.70 (0.64-0.77) | 0.76 (0.65-0.89)** | 0.82 (0.52-1.28) | 0.83 (0.71-0.97)* | 0.76 (0.60-0.97)* |
| Other^1^ | 0.71 (0.56-0.90)** | 0.82 (0.74-0.91)** | 0.91 (0.79-1.04) | 0.86 (0.58-1.30) | 1.00 (0.86-1.16) | 0.92 (0.72-1.16) |
| **Ethno-racial identity** |  |  |  |  |  |  |
| Non-racialized (*ref.*) | 1.00 | 1.00 | 1.00 | 1.00 | 1.00 | 1.00 |
| Racialized | 1.33 (1.02-1.72)* | 1.23 (1.12-1.35)** | 1.56 (1.34-1.80)** | 1.94 (1.52-2.47)** | 1.26 (1.10-1.43)** | 1.46 (1.23-1.74)** |
| **Weekly spending money** |  |  |  |  |  |  |
| $0 or ‘don’t know’ (*ref*.) | 1.00 | 1.00 | 1.00 | 1.00 | 1.00 | 1.00 |
| $1-20 | 0.82 (0.60-1.11) | 0.91 (0.81-1.03) | 0.87 (0.74-1.03) | 0.93 (0.74-1.16) | 0.91 (0.82-1.01) | 1.07 (0.94-1.23) |
| $21-100 | 0.70 (0.53-0.92)* | 0.91 (0.83-1.01) | 1.00 (0.88-1.14) | 0.82 (0.64-1.05) | 0.94 (0.85-1.04) | 1.06 (0.93-1.20) |
| $100+ | 0.82 (0.66-1.03) | 0.95 (0.87-1.03) | 0.95 (0.83-1.10) | 0.71 (0.57-0.88)** | 1.03 (0.94-1.14) | 1.15 (1.02-1.30)* |
| I: Predicts the log-odds of missing MVPA data.  II: Predicts the log-odds of missing CESD-R-10 score.  III: Predicts the log-odds of missing GAD-7 score.  *Note. ref.* = reference category; aOR = adjusted odds ratio; CI = confidence interval. All models control for province and school clustering.  ^1^Includes Secondaire I and II in Québec.  * *p* < 0.05  ** *p* < 0.01 | | | | | | |
